# Multiclass Classifier based Cardiovascular Condition Detection Using Smartphone Mechanocardiography

**DOI:** 10.1038/s41598-018-27683-9

**Published:** 2018-06-19

**Authors:** Zuhair Iftikhar, Olli Lahdenoja, Mojtaba Jafari Tadi, Tero Hurnanen, Tuija Vasankari, Tuomas Kiviniemi, Juhani Airaksinen, Tero Koivisto, Mikko Pänkäälä

**Affiliations:** 1 University of Turku, Department of Future Technologies, Turku, Finland; 2 University of Turku, Faculty of Medicine, Turku, Finland; 3 Turku University Hospital, Heart Center, Turku, Finland

## Abstract

Cardiac translational and rotational vibrations induced by left ventricular motions are measurable using joint seismocardiography (SCG) and gyrocardiography (GCG) techniques. Multi-dimensional non-invasive monitoring of the heart reveals relative information of cardiac wall motion. A single inertial measurement unit (IMU) allows capturing cardiac vibrations in sufficient details and enables us to perform patient screening for various heart conditions. We envision smartphone mechanocardiography (MCG) for the use of e-health or telemonitoring, which uses a multi-class classifier to detect various types of cardiovascular diseases (CVD) using only smartphone’s built-in internal sensors data. Such smartphone App/solution could be used by either a healthcare professional and/or the patient him/herself to take recordings from their heart. We suggest that smartphone could be used to separate heart conditions such as normal sinus rhythm (SR), atrial fibrillation (AFib), coronary artery disease (CAD), and possibly ST-segment elevated myocardial infarction (STEMI) in multiclass settings. An application could run the disease screening and immediately inform the user about the results. Widespread availability of IMUs within smartphones could enable the screening of patients globally in the future, however, we also discuss the possible challenges raised by the utilization of such self-monitoring systems.

## Introduction

Cardiovascular diseases (CVD) are the leading cause of death globally, causing at least 17 million deaths in 2010, representing 30% of all global deaths. According to American heart association (AHA) statistics, almost 80% of these deaths are due to coronary heart disease (CAD) and cerebrovascular disease, leading to sudden heart attacks and brain strokes^1^. European society of cardiology (ESC) report in 2012 also estimates 4 million death in Europe due to CVD, causing 40% of all death in European Union (EU)^2^.

Self-monitoring is a new strategy enabled by employing the generally available smartphones for the screening of CVD. As such, the detection and prevention of CVD could be improved. Smartphones can also be used for the follow-up of patients after cardiac operations to reduce the risk of complications^3–5^. The traditional and existing tools to detect most common CVD such as atrial fibrillation (AFib), myocardial infarction (MI), heart failure etc. within hospitals are usually through electrocardiography (ECG), echocardiography, and other advanced medical procedures^6–8^, which require health professionals to interpret results and identify heart disorders.

New mobile/portable technologies have the potential to streamline detection and prediction of CVDs by enabling individuals to monitor themselves via advanced devices such as mobile phones^9^, smart watches^10^, weighting scale^11^, etc. Several validated mobile/wearable devices are now available which are based on different measuring technologies including: mobile ECGs such as AliveCore Kardia^12^, Zenicor EKG^13^, and Mydiagnostick^14^, Zio Patch^15^, Medtronic implantable loop recorders and wearable SEEQ™ mobile cardiac telemetry^16^; iPhone photoplethysmographs (PPG)^9^; and Microlife A200 and Omron M6 blood pressure monitors for AFib detection^17^. Other approaches have been recently introduced to utilize smartphones/wearables for detection of CVD conditions in a variety of ways as well^4,18–25^. Generally in such diagnosis systems external or internal sensors are used to detect haemodynamics and other physiological parameters of interest. This information is then transmitted to the smartphone using Bluetooth or other appropriate wireless technology^23^. More recent approaches use optical signal–from a fingertip using the built-in camera lens–along with other wearable sensors for autodecetion of cardiac abnormalities^9,24,26,27^.

In this study, we present a smartphone mechanocardiography (MCG) based solution for autodetection of AFib and ischemic conditions by considering only mechanical signals through joint seismocardiography (SCG)^28^ and gyrocardiography (GCG)^29^ signals obtained from built-in accelerometer and gyroscope sensors in smartphones. The use of external accelerometers and (internal) smartphone-based accelerometers in overall physical activity and specifically heart condition monitoring is already established in medical research^30^. A considerable amount of literature has been published on conventional ballistocardiography (BCG) and SCG methods addressing that the mechanical signals have great potential in allowing proactive and non-invasive cardiac performance assessment, e.g. heart arrhythmia, myocardial ischemia, cardiac resynchronization therapy, and heart failure^31^. Additionally, recent investigations report promising findings that support the feasibility of using prospective implantable devices–based on accelerometer and gyroscope sensors–to continuously monitor left ventricular function^32–35^. With the advances in microelectromechanical systems (MEMS) manufacturing, the incorporation of IMUs within wearables/smartphones devices became more common and feasible. This allows easier self-detection to which can potentially improve early diagnosis of heart disease leading to timely interventions and subsequently less medical complications. Our envisioned smartphone app is able to categorize normal condition (regular cardiac motion with sinus rhythm), AFib (irregular motion patten with random rhythm), and ischemic heart disease (abnormal cardiac motion with possibly regular/irregular rhythm). The reason for pursuing this research is that mechanical signals pose potentially valuable information related to the heart performance not obtainable by ECG. More precisely, our hypothesis is that cardiogenic mechanical alterations due to CVD conditions are recognizable using advanced machine learning techniques and thus our research may also provide new venues in self-monitoring for cardiovascular condition detection and care using smart devices. The presented work also facilitates adoption of smartphone cardiography for CVD detection. This paper is a continuation work to the previous investigations in which we considered potential feasibility of SCG-GCG methods for AFib and ischemia detection^36–38^.

## Related Works with ECG and MCG for Cardiovascular Monitoring

### Atrial fibrillation (AFib)

Atrial Fibrillation is a very common cardiac rhythm abnormality, where the atria fail to contract in a coordinated manner, instead vibrating approximately 400 to 600 times (atrial activity) per minute. In this case, contraction of the chambers is irregular and may vary from 40 to 180 times per minute^39^. ECG is the gold standard method for AFib detection. However, AFib can be detected with others techniques as well. A systematic review and meta-analysis on the accuracy of methods for diagnosing AFib using electrocardiography is available in^7^. Another recent review on advances in screening of AF using smartphones has been given in^40^. For instance, Lee *et al*.^9^ primarily used an iPhone 4S to measure a pulsatile photoplethysmogram (PPG) signal in order to detect AFib episodes by recording smartphone’s videocamera. The signal was obtained by a recording made with smartphones’s own videocamera. Recently, we presented a primary solution based on time-frequency analysis of seismocardiograms to detect AFib episodes^36^. The proposed method relies on linear classification of the spectral entropy and a heart rate variability index computed from the SCG signals. In continuation of that study, we developed an extensive machine learning solution^37^ to detect AFib by extracting various features from GCG and SCG signals obtained by only smartphone inertial sensors. This smartphone-only solution for AFib detection showed an accuracy of 97.4%.

### Coronary artery disease (CAD)

Coronary artery disease refers to accumulation and inflammation of plaque in coronary arteries that could lead to heart attack. With ischemic disease, the blood flow to the heart’s muscle is decreased as the coronary arteries are gradually narrowed due to plaque formation within the walls. The majority of myocardial infarctions and strokes result from sudden rupture of atherosclerotic plaques^41^.

The editorial^6^ has mentioned numerous approaches to CAD diagnosis by analysis of ECG depolarization. For example, Abboud *et al*.^42^ proposed high-frequency analysis of electrocardiogram to assess electrophysiological changes due to CAD. As such, high-frequency changes in ECG QRS complex components, also known ans Hyper-QRS, has been considered a sensitive indicator of acute coronary artery occlusion^43,44^. Many other techniques have been also developed to detect acute ischemia using ECG^8,18,19,21,22^. ECG QT-wave dispersion was investigated as a measure of variability in ventricular recovery time and a possible measure for identifying patients at risk of arrhythmias and sudden death after infarction^8^. Myocardial dispersion, also known as strain rate variations, is measured by echocardiography and reflects the heterogeneity of myocardial systolic contraction and can be used as an indicator for susceptibility to arrhythmias in different heart disease groups such as heart failure, ischemia, and infarction^45–47^. In recent years, machine learning algorithms based on wavelet transform feature engineering, pattern recognition, and support vector machine classifier have also been suggested to diagnose CAD conditions^24,48^.

Ischemia can be classified into two major categories according to the presence of the ST segment elevation in ECG. If heart’s major arteries are completely obstructed, the amplitude of the observed elevation is directly linked to the severity of acute or threatening damage to the heart muscle. This type of heart attack is called ST-elevation myocardial infarction (STEMI). For patients with suspected myocardial infarction, but without ST-segment elevation in ECG (only partially blocked coronary arteries), the ECG findings are non-specific and investigation of cardiac markers (e.g. troponin) is required to confirm the diagnosis^49^. In the other category so-called NSTEMI (Non-ST elevation myocardial infarction), the symptoms might be milder or often vague so that other advanced diagnostic methods are considered.

In this paper, we consider multi-class classification of various heart conditions using a smartphone-only solution based on SCG and GCG. We believe abnormal morphological changes in cardiogenic vibrations – possibly due to hypoxic myocardium tissue – are recognizable and therefore can improve detection of heart arrhythmia and ischemic diseases. A potential impact of this research is efficient prevention and follow-up of patients with various heart conditions, enabled by mobile technology.

**Figure 1:**
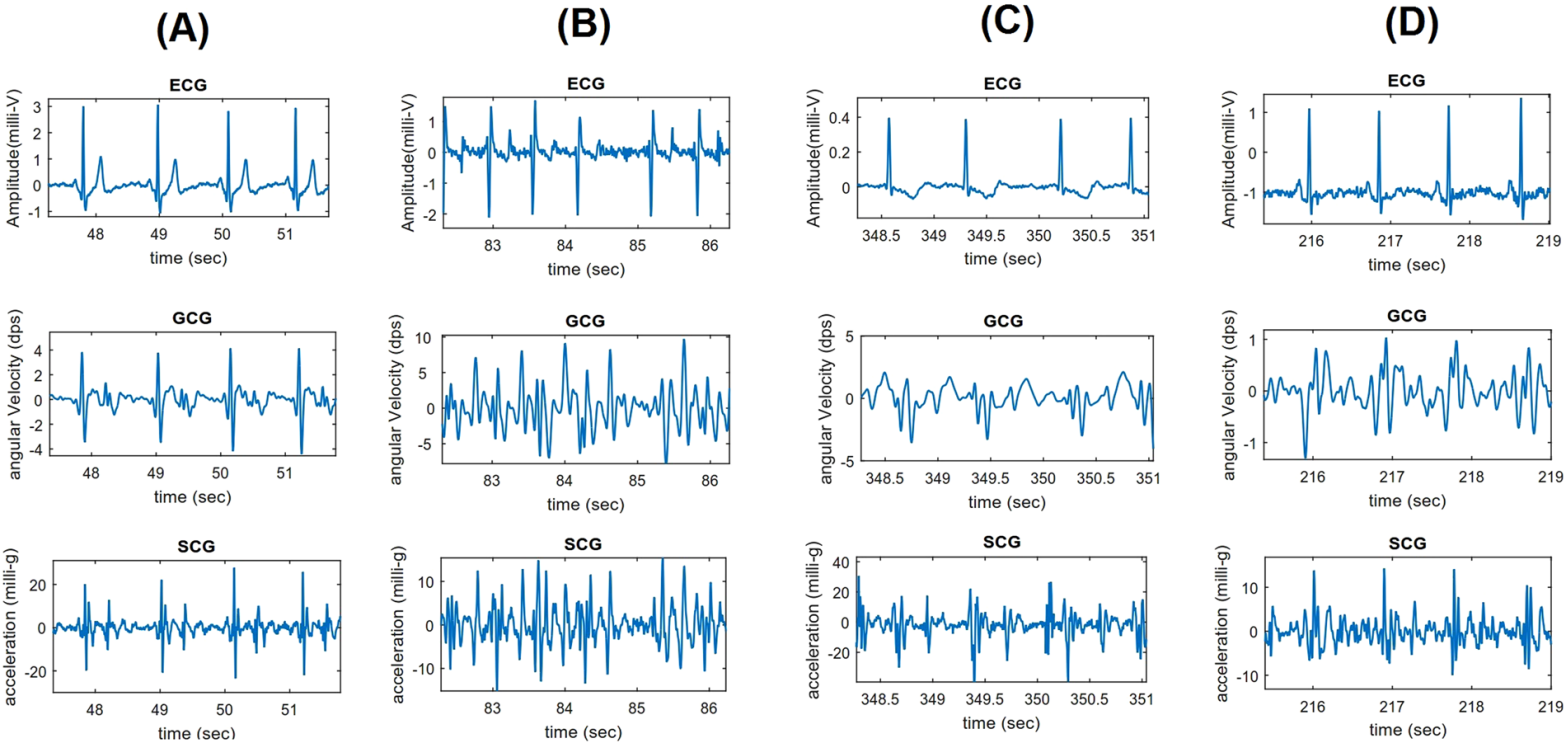
Overall waveform characteristics of normal (**A**), atrial fibrillation (**B**), and coronary artery disease with ischemic changes: T-wave inversion (**C**) and ST segment depression (**D**) conditions shown in ECG (lead I), GCG, and SCG signals.

Figure 1 shows ECG-SCG-GCG cardiac waveform characteristics in normal, AFib, and CAD conditions. As shown, with normal condition both electrical and mechanical signals follow regular rhythm and monomorphic repeating patterns while in AFib condition cardiac signals appear irregular in terms of rhythm and morphology. More precisely, due to the atria failure in mechanical function left and right ventricles may response with abnormal systolic-diastolic functioning. In CAD situation, although regular rhythm is visible in SCG-GCG, cardiac motion pattern has undergone considerable changes such as poor contractility (amplitude reduction), larger diastolic activity, and widened systolic complex (as shown in D multiple and wide wavelets are visible in the onset of systole), potentially due to the artery blockage.

## Human Study Protocols and Demographics

### Data Acquisition and Measurement Protocols

Short recordings (up to 3 minutes) with Sony Xperia Z series smartphones were captured from 23 healthy individuals (all males), 40 AFib patients (22 males, 18 females) verified against simultaneous ECG, 21 non-acute CAD patients (13 males, 8 females) who underwent elective percutaneous coronary intervention (PCI), and 21 myocardial ischemia patients (12 males, 3 females, 6 without demographic information) with acute infarction and ECG changes such as ST-elevation which exclusively refers to these patients. The patient was asked to lie on supine position and either a trained nurse or doctor took care of the measurement by placing the smartphone on the chest (on sternum bone) of the patient. The measurements were conducted according to the Declaration of Helsinki guidelines at Heart Center, Turku University Hospital, Finland with the permission of Ethical Committee of the Hospital District of South-Western Finland. Written informed consent was obtained from all patients. The measurements were short to minimize potential discomfort to the patients. The measurements taken from healthy control individuals were captured from voluntary participants among the University of Turku campus.

### Inclusion and exclusion criteria

The inclusion criteria to the study was that the patient’s ages were at or above 18 years, and that he/she was willing to participate to the study and that they were legal representatives of themselves. Patients suffering from severe memory problems were excluded from the study. Another exclusion criteria was that whether in the investigator’s opinion they might suffer from some other condition that might jeopardize their optimal participation to the study. Due to the availability of suitable patients in the given time interval to conducting the data gathering, the AFib patients, for instance, may have suffered also from other conditions (such as heart failure). Although it might somehow affect to the analysis, we consider that it will not bias the results towards other unknown factors.

### Patient demographics

The demographics of different CVD patient groups (registered) including AFib, coronary artery disease, and acute myocardial infarction are presented in Table 1. Demographics of unregistered patients with missing information are not reported in this table.

**Table 1:** Demographic information of registered study subjects.

Study Group	Age (years) (Min-Max, Mean ± STD)	Height (cm) (Min-Max, Mean ± STD)	Weight (kg) (Min-Max, Mean ± STD)	BMI (kg/m^2^) (min-max, Mean ± STD)
Control (*n = 23)	23–53, 31.4 ± 8	172–190, 180 ± 5	61–125, 82.4 ± 16	20.5–39, 25.5 ± 4
Atrial Fibrillation (n = 27/40)	44–89, 73.3 ± 10	150–193, 171.4 ± 11	45–127.5, 81.5 ± 18	20–39, 27.5 ± 4
Myocardial Ischemia (n = 11/21)	40–83, 65.6 ± 14	150–190, 174.5 ± 12	55–105, 72.3 ± 16	17–30, 24.7 ± 4
CAD (n = 11/21)	58–82, 71 ± 8	154–186, 173.3 ± 10	65–131, 86.5 ± 19	21–38, 29 ± 5

*Number of patients with registered demographic information in each group.

## Feature Extraction and Classification

### Pre-Processing

Each measurement is first pre-processed with a brick-wall fast Fourier transform filter to remove baseline wandering and noise components of the signals within the frequency bands 1–40 Hz. Furthermore, an additional bias and breathing removal step is applied to each measurement axis by subtracting a convolved version of the signal from the original signal as following:

Assuming that the mechanical signal segments *s*(*t*) consist of three additive components as

1 $${\rm{s}}({\rm{t}})={{\rm{s}}}_{{\rm{h}}}({\rm{t}})+{{\rm{s}}}_{{\rm{b}}}({\rm{t}})+{\rm{n}}({\rm{t}}),$$

where *s*_*h*_(*t*) is the precordial vibration signal segment of interest induced by the heart motion, *s*_*b*_(*t*) corresponds to the respiration component, and *n*(*t*) includes all the other residual inertial components and noise. The above described bandpass filtering process significantly eliminates the power of the third component *n*(*t*). Thus it can be presumed to be negligible in comparison to *s*_*h*_(*t*) and *s*_*b*_(*t*).

The effect of breathing component *s*_*b*_(*t*) was then reduced by subtracting an estimate of the breathing from the signal segment *s*(*t*). The estimated breathing signal was obtained by applying a mean filter to *s*(*t*) (uniform normalized all-ones filter of length 50 samples in approximately 200 Hz sampling frequency). The final approximated cardiac signal segment is then given by

2 $${\hat{{\rm{s}}}}_{{\rm{h}}}({\rm{t}})={\rm{s}}({\rm{t}})-{{\rm{Mean}}}_{50}({\rm{s}}({\rm{t}})).$$

### Candidate Features

Next we explain the features used to detect AFib first, and then move onto other features to recognize abnormal waveform of ischemic cases. A key step in the development of machine-learning system is the definition and extraction of the candidate features that highlight the best discrimination between different classes. For the characterization between AFib and sinus rhythm (SR), we define 5 main groups of features based on heart rate (HR), heart rate variability (HRV), spectral entropy (SP-Ent), approximation entropy (AP-Ent), and turning-point ratios (TPR). Intuitively, we consider linear and non-linear behaviour of the measured signal and heart rate variations in both time and frequency domains. It is necessary to characterize beat-to-beat intervals by using a robust technique capable of estimating cycle lengths or correspondingly instantaneous heart rates (IHR) within an acceptable rate of accuracy. We obtain IHRs by using a previously introduced technique, called short-term autocorrelation^36^, to estimate the periodicity of the signals. Moreover, we employ energy features and 1-dimensional local binary pattern (LBP) features which are often used to represent texture or pattern structure found in a signal. Texture analysis resembles human vision and can be used to find abnormal pattern of cardiogenic waveforms in SCG-GCG signals.

### Feature vector generation and majority voting

As in our previous study^37^, we first divide each measurement into shorter segments (of length 10 second), which are then used for the construction of the feature vector. In particular, a feature vector is calculated using data from each of the six axes *(AccX, AccY, …, GyroZ)*. The final feature vector of a single segment is a concatenation of the feature vectors derived from each axis. This is used as input to two-class or multi-class Kernel Support Vector Machine (KSVM) and Random Forest (RF) classifiers. In particular, when using all classes (Normal, AFib, CAD, STEMI) in the multiclass classification setting, all the presented features are selected to the feature vector. The final feature vector length is in that case 265 * 6 i.e. 1590 - for a single 10 second segment. This constitutes of 18 AFib features (1 AP-Ent, 11 TPR, 1 RRI-TPR, 1 SP-Ent, 1 HR, 3 HRV), 11 energy features and 4 uniform local binary patterns (LBP) histograms of length 59. The LBP histograms are formed by applying different spacing between the bits (of 3, 21) and using the same two spacing with Matlab’s cumtrapz integrated version of the input signal. In the case of three classes (Normal, AFib, CAD), all features except the energy features are used. In our classification considerations, Normal class means regular rhythm and pattern, AFib class delineates irregular rhythm and fully random patterns, and CAD class reflects regular rhythm with abnormal morphological patterns.

**Figure 2:**
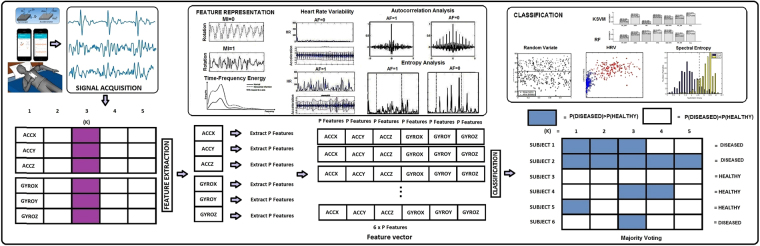
Overall diagram of the machine learning pipeline. Segmented SCG-GCG data are fed to the feature extraction function which forms a row-wise concatenation of features corresponding to each axis. In classification part, the final models are cross-validated by class prediction for each of the test cases is the dataset.

As in^37^ we report most of the results with majority voting, which means that all the 10 second segments of a particular measurement (person) are used to vote for the final class. In the multiclass framework, this simply means that the class which is the most common among the evaluated segments is chosen to be the final result. In the two-class case, the class which is more common is also chosen as the detection result. The confusion matrix reported without majority voting consists of all 10 s segments which have been evaluated. Figure 2 shows our machine learning pipeline for cardiac condition detection using smartphone MCG data.

### Heart Rate Estimation

We consider short-term autocorrelation (AC) algorithm^36^ which is able to analyse the periodicity of a signal in the segmented samples. We calculate the short-term AC by first segmenting the acquired signals–from each channel of accelerometer and gyroscope sensor–into 10 s episodes and subsequently divide each episode into sub-segments with the duration of 2.5 second. Since the smartphone sampling frequency (*F*_*s*_) is tuned to approximately 200 Hz, each episode and sub-segment will contain 2000 and 500 samples, respectively. The segmentation is performed so that the consecutive sub-segments overlap by 1.5 seconds. Therefore, each 10 second episode consists of eight 2.5 second sub-segments, which are all unique but should share at least one heart beat with the neighbouring sub-segment.

Assume that a 2.5 second sub-segment *u*(*t*) is chosen within signal *s*(*t*). If *u*(*t*) encompasses only two heartbeats, then the cardiac cycle length between the two heartbeats can be measured by calculating the period of *u*(*t*). To this end, the first 1.5 seconds of *u*(*t*) — denoted by *u*_1.5_(*t*) — is cross-correlated with *u*(*t*). This yields

3 $${\rm{R}}({\rm{u}}({\rm{t}}),{\rm{i}})=\sum _{{\rm{j}}}{\rm{u}}({\rm{j}}){{\rm{u}}}_{1.5}({\rm{j}}+{\rm{i}}),$$

where *j* is a discrete variable denoting the time indices, and only positive indices *j* + *i* up to the number of samples in *u*_1.5_(*j*) are taken into account. The inter-beat time intervals can be therefore estimated by locating the first side peak of *R*(*i*). This is accomplished by calculating the index of the first side peak

4 $${{\rm{i}}}_{{\rm{first\; peak}}}={arg}_{|{\rm{i}}| > {{\rm{i}}}_{0}}max({\rm{R}}({\rm{u}}({\rm{t}}),{\rm{i}}),$$

where *i*_0_ is a limit, which is set to be *i*_0_ = 1/3*sampling frequency. The limit *i*_*o*_ is chosen with respect to this fact that a period of at least 1/3 seconds in the signal is a sufficient threshold for heart rates below 180 bpm. Thus, the corresponding estimated interbeat duration *RR* is obtained as

5 $${\rm{RR}}={{\rm{i}}}_{{\rm{first}}{\rm{peak}}}/{{\rm{F}}}_{{\rm{s}}}.$$

The algorithm can subsequently return eight cardiac cycles (either *R* – *peak* to *R* – *peak* or SCG-GCG dominant *Peak* to *Peak*) from one signal segment (of length 10 seconds) which are denoted as RR _*k*:1−8_. Heart rate (HR) is estimated as MEDIAN(RR_*k*:1−8_).

### Heart Rate Variability

The first set of generated features consists of heart rate (HR) and HRV indexes in time-domain. We collect three HRV features (HRV_1_, HRV_2_, and HRV_3_) derived by applying certain operators directly on the series of successive cardiac time intervals. To compute these HRV features, we first define the *k* th RR-interval–obtained by short term AC–by

6 $${{\rm{RR}}}_{{\rm{k}}}={{\rm{R}}}_{{\rm{j}}+1}-{{\rm{R}}}_{{\rm{j}}},$$

where R_*j*_ denotes timing of the *j*th heartbeat. HR is calculated as the median of the RR_*k*:1−8_ and HRV_1_ is calculated as *median absolute difference*(MEAD) of these successive cardiac intervals. Assuming that the derived *RR* intervals are stored in the vector *x*, the *MEAD*(*x*) is obtained by

7 $${{\rm{HRV}}}_{1}={\rm{MEAD}}({\rm{x}})={\rm{median}}|(R{R}_{k\mathrm{:1}-8})|,$$

where operator *median* returns the median value of the first order differences^37^. We consider the median value instead of mean as it is tolerant to outliers. Furthermore, we calculate two higher order HRV indexes, denoted respectively as HRV_2_ and HRV_3_, by

8 $${{\rm{HRV}}}_{2}={\rm{median}}(|Diff\mathrm{\ (}R{R}_{k\mathrm{:1}-8})|),$$

where HRV_2_ returns the *median* value of the second order differences between the consecutive elements, and

9 $${{\rm{HRV}}}_{3}=\text{median}(|Diff\mathrm{\ (|}R{R}_{k\mathrm{:1}-8}|)|),$$

returns the median value of the absolute value of the second order differences^37^.

### Approximate Entropy

Approximate entropy (AP-Ent) is a popular approach in analysing the complexity of the time series. Namely, AP-Ent is a self-similarity parameter that quantifies the unpredictability of fluctuations in a time series. AP-Ent considers the probability that particular patterns of observations will not be followed by extra similar observations^50,51^. With calculating AP-Ent, time series containing regular patterns such as sinus rhythm are expected to have a relatively small AP-Ent, while a less predictable or irregular pattern signal (e.g. *AFib*) should have a higher AP-Ent index^50^. The details of our calculation of AP-Ent can be found in^37^.

### Spectral Entropy

Spectral entropy (SP-Ent) has been known as a measure of uncertainty or in other words randomness of a time series. SP-Ent is a tool to quantify the spectral complexity of a signal^52^. SP-Ent relies on power spectral density (PSD) analyses *P*(*f*) which is commonly obtained by fast Fourier transform (FFT). The PSD *P*(*f*) is density function which aims to show the distribution of power as a function of frequency^52^. We limit the frequency band *f* to [1–11 Hz]. In order to minimize the effect of frequent low frequency components, an estimated noise floor is discarded from *P*(*f*) by filtering frequencies with energy amplitude $P(f) < \frac{1}{6}\times \,{\rm{\max }}(P(f))$. The resulting spectrum is then normalized to unit probability as

10 $${{\rm{P}}}_{{\rm{n}}}({\rm{f}})={\rm{P}}({\rm{f}})/\sum _{{\rm{f}}}{\rm{P}}({\rm{f}}).$$

This normalization is necessary, as it essentially considers the frequency spectrum as a probability distribution prior to computing of spectral entropy. Finally, the spectral entropy of the signal *P*_*n*_(*f*) is computed as

11 $$\text{SP} \mbox{-} \text{Ent}=-\,\sum _{{\rm{f}}}{{\rm{P}}}_{{\rm{n}}}({\rm{f}}){\rm{l}}{\rm{o}}{\rm{g}}({{\rm{P}}}_{{\rm{n}}}({\rm{f}})).$$

The computed *SP*-*Ent* for samples containing more random frequency components, e.g. *AFib*, is greater than samples with less randomness, e.g. sinus rhythm. In other words, the larger the *SP*-*Ent*, the more random the signal frequency component, which implies that an aperiodic signal may have higher randomness level (*SP*-*Ent*) than a periodic. Therefore, the rate of randomness is used as an individual feature for distinguishing periodic or aperiodic segments in cardiac motion signals^37^.

### Turning-point Ratios

Turning point ratio (TPR) is a non-parametric statistical approach to determine the randomness in a time series. We define operator RD to derive the total number of consecutive increasing and decreasing runs in signal segment *x*. TPR of *x* is therefore defined as

12 $${\rm{TPR}}({\rm{x}})=\frac{{\rm{RD}}({{\rm{FIL}}}_{{\rm{m}}}({\rm{x}}))}{{\rm{N}}-2},$$

where N is the number of samples in *FIL*_*m*_(*x*). We consider turning point ratios in both input signal denoted as *TPR*(*x*) and obtained RR time interval series from the same segment defined as *RRITPR* = *TPR*(*RR*_*k*:1−8_). In general, we extract a total of 11 TPR-based features from the original input signal by means of passing it into different filtering (FIL) schemes, including various frequency bands, to retrieve waveform complexity information of the input signal (see the details in^37^).

### Energy Features

We use a total of 11 energy features derived from each signal segment specific to an axis. The features are derived by calculating the energy i.e. by summing the pre-processed and squared signal and by dividing it with the length of the signal segment. We consider 11 different filtering bands (FIL in the equation) as well as 10 s length signal (*F*_*s*_ = 200 Hz) segments (in total 2000 samples)

13 $${{\rm{ENE}}}_{{\rm{m}}}=\sum _{{\rm{i}}=1}^{2000}{{\rm{FIL}}}_{{\rm{m}}}({\rm{signal}}{({\rm{i}})}^{2})/2000$$

The individual energy features *m*, (*m* = 1..11) contain the energy of the signal in different frequency sub-bands, each band corresponds to a certain frequency spectrum. Furthermore, some features are passed through an absolute value operation and a long triangular shaped smoothing filter^37^.

### Local Binary Patterns

As new features we consider the Local Binary Patterns (LBPs), which have been successfully used in image processing applications such as texture analysis, segmentation and feature detection^53^. The idea behind 2 Dimensional-LBP is based on evaluating the (intensity, differences, etc… of) neighbourhood pixels found at certain angles when we rotate from 0–360 degrees in anti-clockwise direction. The neighbourhood pixel coordinates around a point *g*_*c*_ are found by (−*Rsin*(2*πp*/*P*), *Rcos*(2*πp*/*P*))

14 $${{\rm{LBP}}}_{{\rm{P}},{\rm{R}}}=\sum _{{\rm{p}}=0}^{{\rm{P}}-1}{\rm{s}}({{\rm{g}}}_{{\rm{p}}}-{{\rm{g}}}_{{\rm{c}}}){2}^{{\rm{p}}},$$

where *g*_*c*_ and *g*_*P*_ are respectively values of the central point C, and the surrounding point P in the circle neighbourhood with a radius R, and function s(x) is defined as:

15 $${\rm{s}}({\rm{x}})=(\begin{array}{l}1,{\rm{x}}\ge 0\\ 0,{\rm{x}} < 0\end{array}$$

Those coordinates which do not exist are assigned a value using interpolation. Apart from being faster to calculate, rotation invariance is another well known property of the LBPs^53^. An effcient variant of 2D-LBP called uniform 2D-LBP which includes only those binary patterns which change only once, either from 0–1 or from 1–0. Uniform patterns have minimum transitions and as such act as pattern templates for interesting features in an image. These uniform LBPs are thought to cover fundamental properties of most textures observed in a neighbourhood around a center point^53^.

In our application we have used 1D-LBPs variant called 1-dimensional uniform LBP^54^. 1D-LBP is suitable for our input vector *v* data type. For any time index in the input vector the neighbourhood we consider is, *d* pixels (samples) before and after the index position being analyzed. An additional parameter called spacing is also used to speed up the computation and to extend the used local neighborhood. Given *P* neighbours (eight in our case), for all the elements in the input vector we calculate the LBP for the time index *i* using the formula:

16 $${{\rm{LBP}}}_{{\rm{P}}}({\rm{v}}[{\rm{i}}])=\sum _{r=0}^{{\rm{P}}/2-1}{\rm{s}}[{\rm{v}}[{\rm{i}}+{\rm{r}}-{\rm{P}}/2]-{\rm{v}}[{\rm{i}}]]{2}^{{\rm{r}}}+{\rm{s}}[{\rm{v}}[{\rm{i}}+{\rm{r}}+1]-{\rm{v}}[{\rm{i}}]]{2}^{{\rm{r}}+{\rm{P}}/2}$$

Each histogram bin of the LBP is updated as many times as there are time instants i (except at the borders of the segment where the neighbors do not exist). We consider only the 8-bit neighbourhood, which have been most commonly used in previous studies. All the possible uniform LBP patterns for 8-bits are found. A smaller subset of histogram for the uniform patterns is also created in which the values (of uniform patterns) from the above created histogram are assigned. We combine all the non-uniform LBPs histogram indices for an extra last bin in the uniform histogram. As the original 8-bit LBP histogram length is 256, it is reduced to 59 with using uniform patterns only. The resulting histogram vector with varying spacing parameter is taken as a feature for the classifier. Different spacing values can be selected to generate uniform LBP histograms covering different sized neighbourhoods.

**Figure 3:**
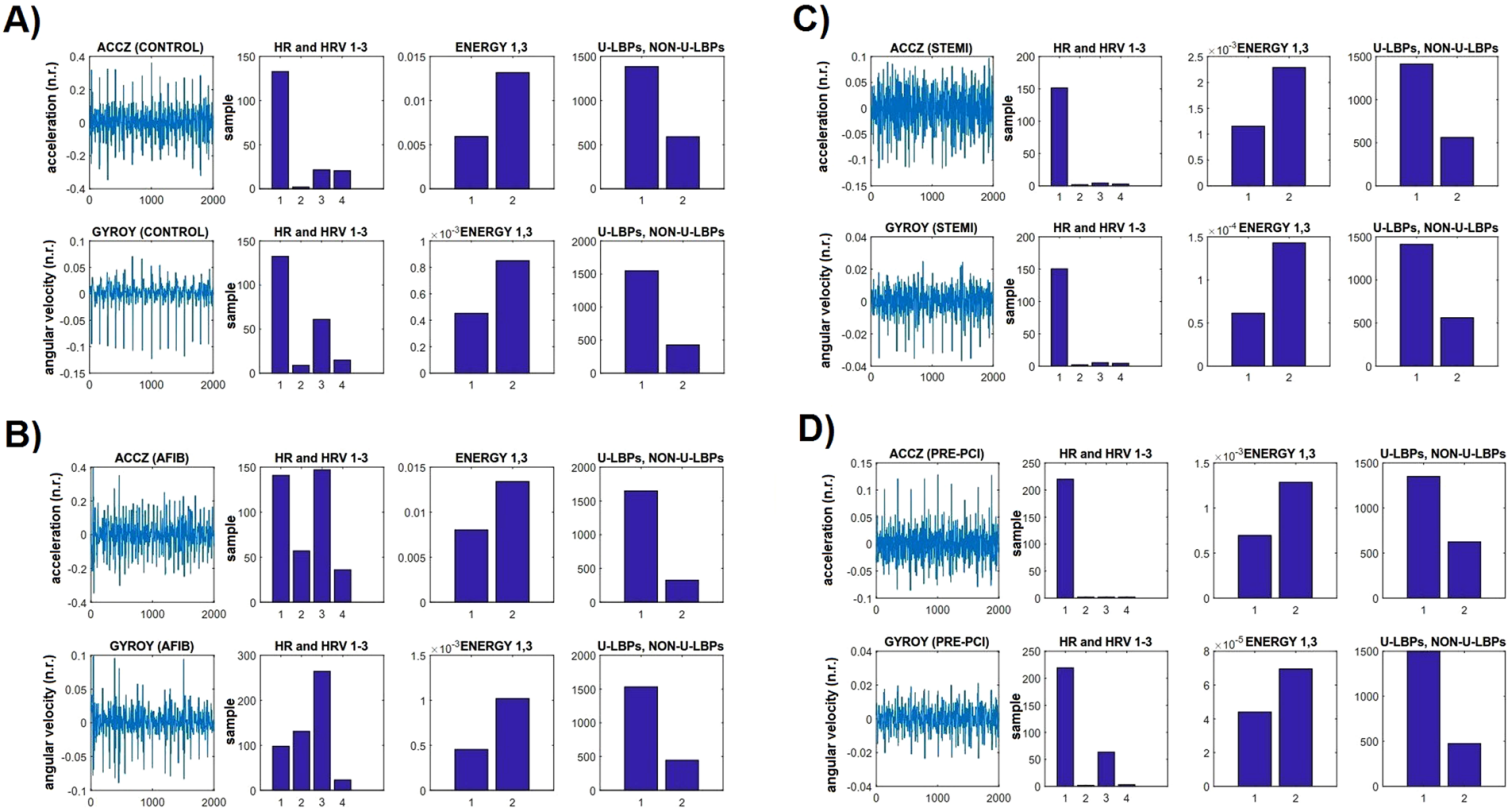
SCG-GCG waveforms and corresponding selected features obtained in normal (**A**), AFib (**B**), STEMI (**C**), and Pre-PCI (**D**) conditions.

Figure 3 shows waveform characteristics of the SCG-GCG and corresponding (selected) features generated for each sensor modality. It can be observed in this case that the magnitude of HRV is increased during AFib, but not during the other conditions. On the other hand, there are changes in the signal energy as well as in the overall number of uniform (and non-uniform) patterns during Pre-PCI and STEMI.

## Experimental results

In our previous study^37^, we used a total of 18 features in a two-class classifier setting to distinguish between AFib and normal persons. Here we extend the previous study by adding two more classes - CAD and STEMI - to the classifier evaluation framework. Keeping these two new classes in mind, we introduced the LBP features in the previous section. The final multiclass classifier will use a combined feature vector of all the 18 AFib features used in^37^ and the LBP features. However, before going to the implementation and evaluation of the multiclass classifier, it is necessary to study the properties of each feature in two-class settings, keeping the two new classes in mind. In the following evaluations, only the features designed in particular to that setting are used. In all tests, we use a leave-one-person-out cross-validation (LOOCV) which is well suited to studies with small or limited amount of samples. We report sensitivity (SE), specificity (SP), and accuracy (ACC) metrics to evaluate classification performances according to the Eqs 1–3 in the supplementary information (SI).

### AFib detection in two-class setting

**Figure 4:**
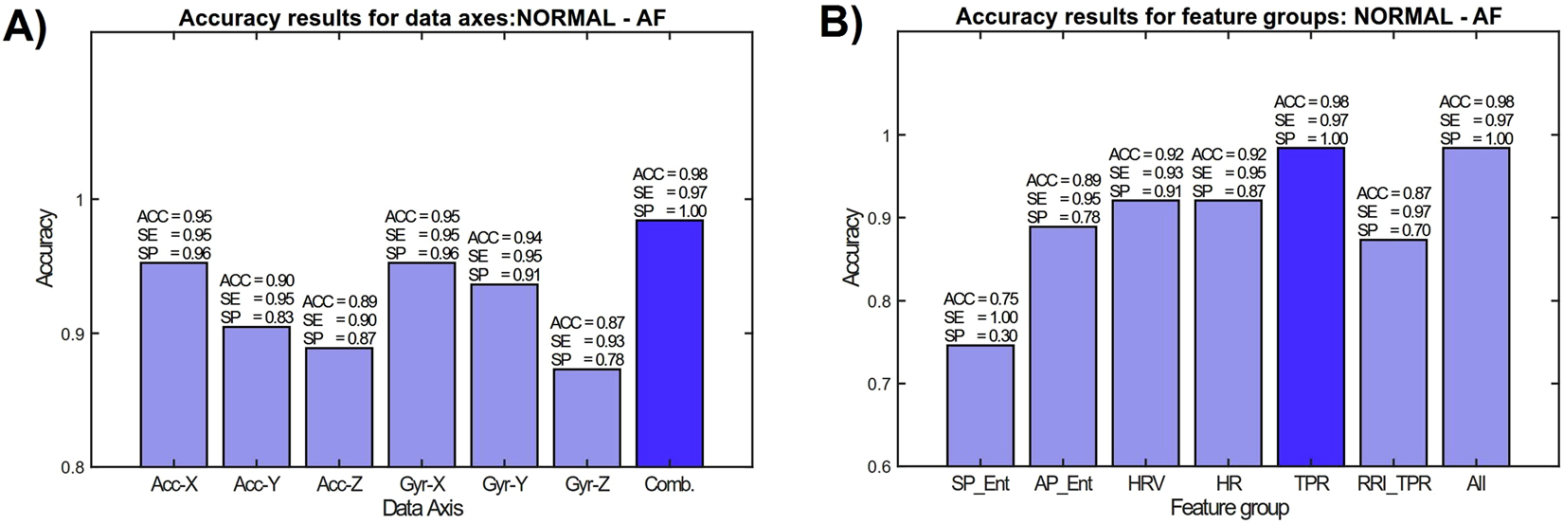
Effect of mechanical axes (**A**) and each feature (**B**) to the overall AFib classification performance.

As an extension to our previous study^37^, where there were 16 AFib patients, we have collected data from up to 40 patients, including the previously collected data. The control group remains the same as in^37^, i.e. there are total of 23 heathy individuals. The overall two-class AFib classification rate with each set of features is shown in Fig. 4. Figure 4(A) shows that the combination of the axes provides the best classification rate with respect to each individual axis. From Fig. 4(B) it can be observed that HRV (heart rate variability) and TPR (turning-point ratio) are the best performing features.

Table 2 represents classification accuracy between AFib and normal sinus rhythm cases. As shown, the best result was obtained with specificity of 100% (Healthy classified as Healthy) and sensitivity of 97.5% (AFib classified as AFib) using KSVM. With RF classifier, same sensitivity but slightly poorer specificity (95.6%) were achieved. Thus, a total accuracy of 98.4% and 96.8% was obtained for KSVM and RF classifiers, respectively. In comparison with^37^, adding the new AFib samples/subjects improved the results.

**Table 2:** AFib detection performance using KSVM and RF with and without majority voting.

AFib	RF		KSVM	
	Without Majority Voting	With Majority Voting	Without Majority Voting	With Majority Voting
ACC (%)	92.0	96.8	94.8	**98.4**
SP (%)	87.6	95.6	94.3	**100**
SE (%)	94.5	97.5	95.0	**97.5**

### Pre-PCI vs. Normal in two class setting

**Figure 5:**
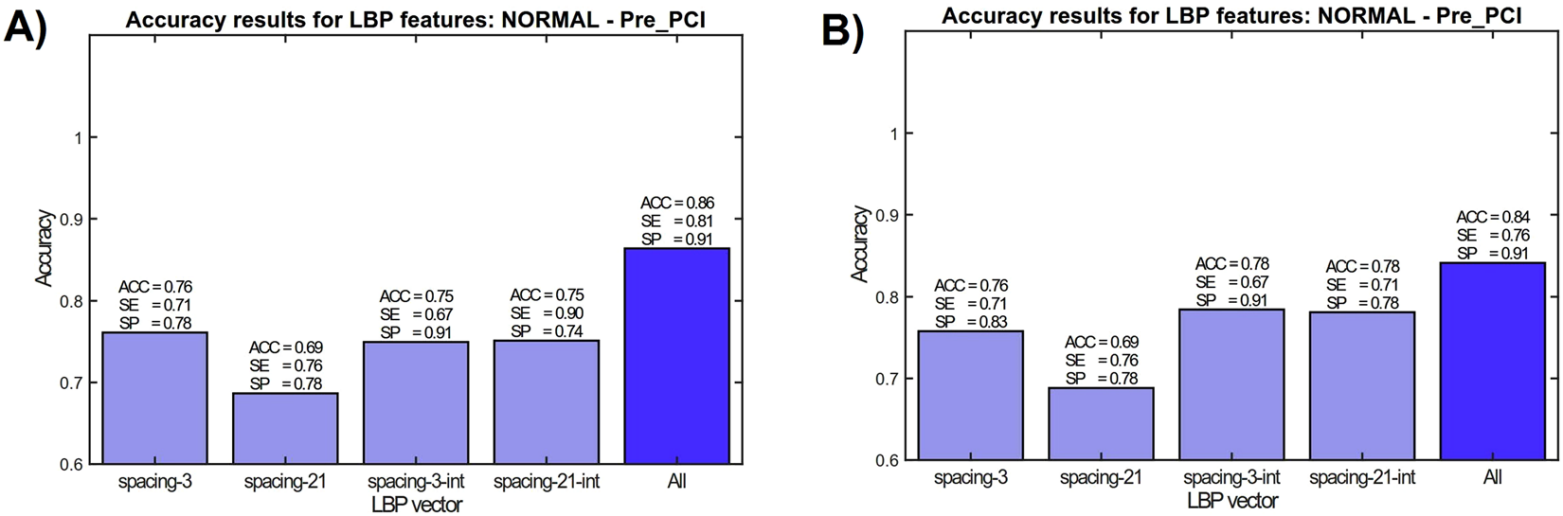
Effect of each feature to the overall classification performance in Healthy vs. Pre-PCI with Kernel SVM (**A**) and random forest classifiers (**B**).

Another group of patients are suspected for myocardial infarction and after clinical considerations were preferred for elective percutaneous coronary intervention (PCI) procedure. Smart phone measurements with these patients were conducted before the PCI and therefore, they are denoted as Pre-PCI cases. The reason to select Pre-PCI patients instead of normal CAD patients was to evaluate the distinction of hemodynamically significant stenosis without ST elevation in the ECG to ST elevation myocardial infarction. Also, as the control group used for evaluation contain quite different demographics in comparison with the other groups, the distinction between the Pre-PCI and STEMI group with regard to demographics should be smaller. In Fig. 5 LBP features are used in Normal vs. Pre-PCI classification. It can be observed that combining the LBP histograms with different granularities (dense and coarse spacings between samples) improves the classification rate, as well as using Matlab’s cumtrapz integration–to convert signals as indicators of cardiac angular displacement (GCG) and linear velocity (SCG). When considering different classifiers and feature vectore in Normal vs. Pre-PCI classification (Fig. 5), it appears that combination of all features improves the overall result with both KSVM (average accuracy 86%, sensitivity of 81%, and specificity of 91.5%) and RF (average accuracy 84%, sensitivity of 76.2%, and specificity of 91.3%). Table 3 reports performance metrics obtained by two separate classifiers in two different conditions.

**Table 3:** Pre-PCI identification performance in two class setting for KSVM and RF.

PrePCI	RF		KSVM	
	Without Majority Voting	With Majority Voting	Without Majority Voting	With Majority Voting
ACC (%)	81.2	84.0	86.0	**86.0**
SP (%)	89.0	91.3	82.5	**91.3**
SE (%)	73.0	76.2	82.0	**81**

### Pre-PCI vs. STEMI in two class setting

**Figure 6:**
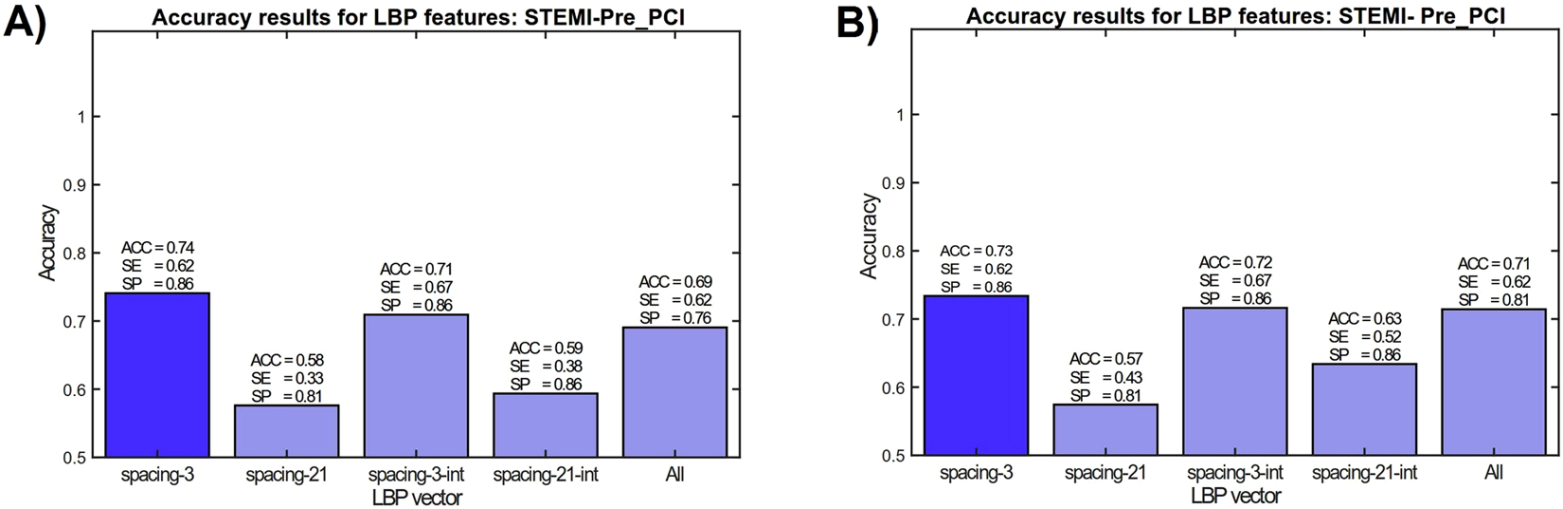
Effect of each feature to the overall classification performance in STEMI vs. Pre-PCI with Kernel SVM (**A**) and random forest classifiers (**B**).

As mentioned, the most relevant individual pair of classes is Pre-PCI vs. STEMI, as it could be expected to be less affected to the differences in the demographics. The effect of different features on STEMI vs. Pre-PCI classification (again, two-class case) is shown in Fig. 6. It can be observed that dense spacing without cumtrapz function provides the best classification rate, while with the combining of axes the detection rate is slightly lower. The overall best sensitivity and specificity of this two-class experiment are 63% and 79% with average accuracy of 71.6%.

The purpose of examining the two-class performances of subsets of features is to evaluate the effect of individual sets of features to the overall classification performance in order to be able to perform multiclass classification effectively. The two-class performance results are summarized in Table 4 with and without majority voting.

**Table 4:** STEMI versus PrePCI detection performance with and without majority voting using RF and KSVM.

STEMI vs PrePCI	RF		KSVM	
	Without Majority Voting	With Majority Voting	Without Majority Voting	With Majority Voting
ACC (%)	**71.6**	71.4	70.6	69.0
SP (%)	**79**	81	79.8	76.2
SE (%)	**63**	62	60.3	62

### Evaluation of the Two-class Classifiers

**Figure 7:**
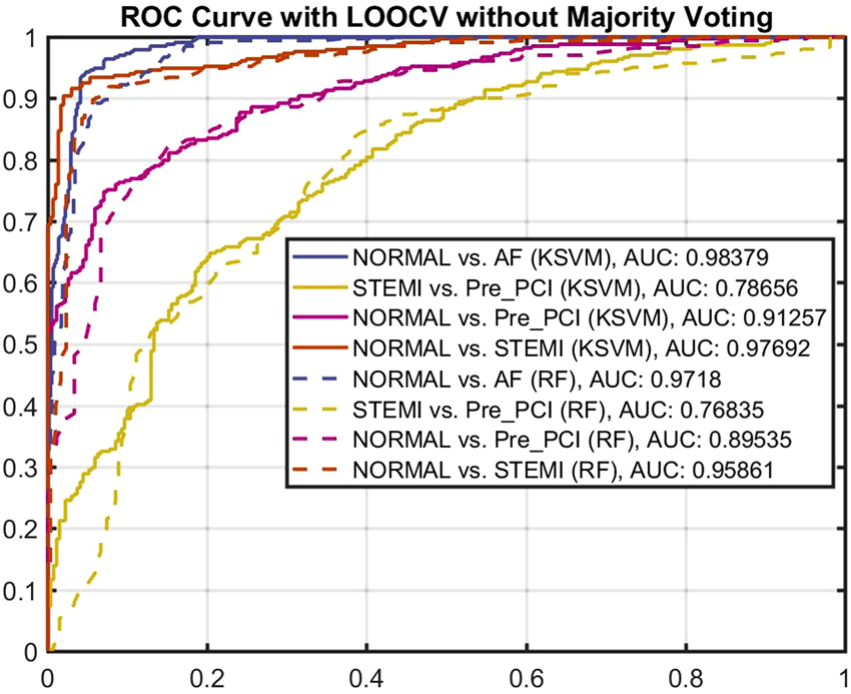
ROC curve showing the classification performance of two-class setting with KSVM and RF.

Figure 7 shows classification accuracy using receiving operator characteristic (ROC) curves obtained for two-class setting using Kernel SVM and RF classification models. As shown, the area under the curve (AUC) obtained by KSVM shows slightly better performance rate as compared to RF in all cases. This implies that our features give robust representation of cardiovascular condition distinguished by two different classifiers in this study. Comparing different CVD conditions, both classifiers were able to detect AFib and CAD conditions with high accuracy. However, they were not able to distinguish ischemic conditions, i.e. STEMI versus Pre-PCI, with diagnostic ability of more than 78%. This is expected as these two conditions are in principle originated from the same phenomena but one belongs to a relatively stable condition for instance NSTEMI, while the other case, STEMI, refers to a crucial and fatal condition. In all cases, classifiers were able to discriminate normal condition from abnormal conditions with high rate of certainty which supports our hypotheses in this study. Tables 1 and 2 in the supplementary document present confusion matrices of two-class case settings with and without majority voting.

### Evaluation of the Multi-class Classifiers

The confusion matrices of multiclass classification cases are shown in Tables 4–6 in the SI document. In particular, we use two test cases for multiclass classifiers; (Normal, STEMI, Pre-PCI) and (Normal - AF- STEMI - Pre-PCI). The first one (3-class) is to test the case of CAD/STEMI detection mainly and the second one (4-class) is to test the overall performance of all data gathered in this study. The accuracy in multiclass settings is calculated as in two-class setting as the sum of the diagonal of the confusion matrix divided by the overall sum of elements within confusion matrix. The accuracy of the 3-class classifier (obtained from the confusion matrix) is 73% without majority voting, and 78.46% with majority voting. The accuracy of the 4-class classifier is 71.17% without majority voting and 75.24% with majority voting. In the multiclass settings using RF classifier the accuracy of 73.9% was obtained in 3-class case. With majority voting the same accuracy of 73.9% was obtained. In the 4-class case the performance of the RF classifier without majority voting was 70.2% and with majority voting 75.2%. Thus, both classifiers were able to separate the classes in each case with more than 70% accuracy.

Complementary to the above performance metrics for multiclass setting, we calculated another metric so-called *F*_1_ score which is an average *F*_1_ value from the classification types. Table. 7 (SI) indicates definition of parameters for scoring. The scoring was based on leave-one-person-out cross validation and the values were achieved according to Equations 1–6 as described in the SI. Accordingly, Tables 5 and 6 represent *F*_1_ scores calculated for 3- and 4-class setting with and without majority voting, respectively. As such, for the 3-class setting the best *F*_1_ score achieved by KSVM without majority voting (78%), while for the 4-class setting the best score was given by RF classifier (74%) again without majority voting.

**Table 5:** RF and KSVM F1 scores for 3-class setting.

F score	Without Majority Voting		With Majority Voting	
	RF	KSVM	RF	KSVM
F1n	0.88	0.91	0.84	0.86
F1m	0.74	0.75	0.75	0.72
F1p	0.56	0.67	0.57	0.56
F1	0.72	**0.78**	0.72	0.72

**Table 6:** RF and KSVM F1 scores for 4-class setting.

F score	Without Majority Voting		With Majority Voting	
	RF	KSVM	RF	KSVM
F1n	0.82	0.89	0.80	0.85
F1a	0.77	0.81	0.75	0.77
F1m	0.65	0.62	0.56	0.57
F1p	0.70	0.60	0.60	0.59
F1	**0.74**	0.73	0.68	0.70

## Discussion

We presented an approach for classifying multiple heart conditions using well known principles of seismocardiography and gyrocardiography with full analysis of signals derived from a 6-axis smartphone built-in inertial sensor. We were able to show, that in multiclass settings the majority voting improved the detection rate, when we used person based LOOCV cross-validation in obtaining the results.

MCG-based cardiac monitoring has pivotal clinical implications as it reliably detects cardiac abnormalities without any additional hardware and provides a new easy-to-use and accessible concept for screening purposes. The findings of this study, while preliminary, suggests that measuring the mechanical movement of the heart muscle offers an entirely new and innovative method to evaluate cardiovascular status. One issue which may affect the results is the fact that control group demographics are quite different from other groups. Although this is not expected to influence to CAD (Pre-PCI) vs. STEMI classification–which is perhaps the most relevant two-class case–it should be taken into account in interpreting the results. It is possible that interpreting the Pre-PCI group as a substitute to traditional healthy control group (with same demographics) can decrease the performance of the classification. On the other hand, it would not be realistic to consider healthy group without any indication of accumulation of atherosclerotic plaque as control group, since it is very unlikely that a healthy person belonging to this group would experience STEMI. Therefore, the only relevant control group are cardiac diseased patients who are at the real risk of having STEMI. Nevertheless, as the patients in the Pre-PCI (and STEMI) group may have suffered from other diseases (e.g. heart failure) as well, it is necessary to perform further studies to confirm our findings with larger sample sizes and more relevant control group settings.

Currently, standard method to establish cardiac disorders is a 12-lead ECG as it determines presence of arrhythmia, conduction defects, ischaemia, and signs of structural heart disease^55^. Our presented MCG monitoring provides a novel way — based on solely measuring mechanical activity — for AFib detection independent of 12-lead ECG and with a comparable diagnostic accuracy of 98%. Strikingly, there is no need to get electro-physiological signals (e.g. ECG) from the heart, but only the precordial vibrations. For STEMI diagnosis, clinical issue with ECG-based methods is the high frequency of false positive ECG findings such as early repolarization as well as ECG findings hindering ischemia detection such as the left bundle branch block, pacemaker rhythm or significant left ventricular hypertrophy. Current computer-aided algorithms for STEMI diagnosis possess a limited sensitivity (of 30–70%) and specificity (of 70–100%)^56^. Although the presented measuring approach revealed inband sensitivity and specificity values, its diagnostic performance for STEMI detection must be analysed not only with ECG data, but when taking clinical symptoms and coronary angiography findings into account. Such a holistic contextual analysis is routine for diagnosing mechanical wall motion abnormalities found in STEMI patients and heart failure patients with reduced ejection fraction (HFrEF).

We discuss some of the main limitations of this work as well. Although we were able to separate the four classes, i.e. Normal, AFib, Pre-PCI and STEMI with a promising accuracy, the underlying physiological mechanism for the separation between the last two classes is still unclear. For AFib and normal classes, it seems, that the separation could be justified by the different heart rate variability resulting from irregular ventricular rate of the AFib, against the Normal class. However, it is possible, that instead of capturing the true physiological meaning of ST-segment elevation in ECG (in the case of STEMI), for instance, the LBP features could just represent the distinction between a shapes of the heart signal for the cases of a stressful event such as STEMI (with acute chest pain), and non-stressful event such as in Pre-PCI in comparison with less “abnormal” cases of sinus rhythm and AFib. As another limitation of this study, although AFib classification was efficient, we did not consider the detection of other arrhythmias, such as atrial flutter (AFL), which should be done in the future. The features used for AFib detection might be, at least partially, usable for AFL detection also, by simply extending the multiclass classifier with AFL class data. The reason for not considering other arrhythmias and premature beats in this study was mainly because of the availability of suitable training data. As a summary, much more work is still needed before any clinically relevant smartphone application could be provided for true patient use. Still, this work is a primary example for demonstrating that in the future an AFib screening application (or equivalent) could be extended to cover a more versatile set of abnormal heart conditions to be detected.

This study illustrates that it is possible to implement a novel classification system possibly aiding in the diagnosis of multiple heart conditions. However, smartphones or their inertial sensors have not been traditionally targeted toward the heart measurements, and necessary further validation and risk assessment must be conducted in order to evaluate real end-use of final system, for example in the form of smartphone application or equivalent. The research conducted further raises some critical ethical issues. For instance, what if a smartphone application or equivalent - despite of possible precautions and instructions - would declare a patient suffering from STEMI as not being in need of instant medical care. Even the most recent smartphones with advanced IMUs are not intended to be used for critical diagnosis whose incorrect (or correct) result might threaten human lives. In fact, the conditions and terms of application stores (such as Google’s Play), explicitly deny using the Apps for critical purposes, whose incorrect operation could cause serious physical injury.

The reasons described above limit the possible commercialization and usage of a system like the one proposed in this paper. Despite this, there already exist multiple solutions in mobile marketplaces for AFib detection, which could perhaps be seen as “less” critical application, since the focus is rather in prevention by early detection, rather than on patients possibly in need of immediate medical care (and where the time-to-treatment critically affects to the outcome of the treatment that the patients receives). Also, there already exists ECG based solutions for patient telemonitoring. However, even with using ECG, blood markers may be used to find further evidence of STEMI. Our future direction in cardiac motion signal processing is to expand our algorithm development with larger group of CVD patients having different cardiac disorders. A randomized clinical trial of CVD screening using smartphones should be carried out to assess reliability and credibility of m-health Apps aiming to detect heart diseases.

In conclusion, this paper addressed the globally important issues of detection of AFib, CAD, and acute STEMI non-invasively with smartphone mechanocardiography. We were able to show that it is be possible to use the built-in inertial sensors of the smartphones to detect some of these conditions, or even multiple conditions, without any external equipment such as ECG leads or wires. Due to global availability of smartphones, it could be possible to integrate a professional diagnosis system as a part of efficient global prevention and detection of heart diseases. However, there is an evident need for further studies such as controlled blinded tests as well as testing the usage of the application in limited distribution in the full supervision of trained medical staff, before any such application could be made available. Although the need for solution described in this paper is evident, there are many important ethical issues and precautions involved, before an actual system could even be tried to be used to reduce the time-to-treatment of real STEMI patients, for instance. In any case, either as a part of telemonitoring system or as a supplement to ECG, inertial smartphone/wearable sensors could potentially be a way to increase the detection performance of heart conditions covered in this paper.

### Data availability

The data that support the findings of this study are available from Turku University Central Hospital (TYKS) but restrictions apply to the availability of these data, which were used under clinical permissions for the current study, and so are not publicly available. Data are however available from the authors upon reasonable request and with permission of TYKS.

## Electronic supplementary material

Supplementary Information
