# Supplementary material for: Multiclass Classifier based Cardiovascular Condition Detection Using Smartphone Mechanocardiography
Source: Sci Rep. 2018 Jun 19;8:9344. doi: 10.1038/s41598-018-27683-9 (PMC6008477; doi:10.1038/s41598-018-27683-9)
Supplement: Supplementary file 1 — Supplementary Information [file 41598_2018_27683_MOESM1_ESM.pdf]

# Multiclass Classifier based Cardiovascular Condition Detection using Smartphone Mechanocardiography

**Zuhair Iftikhar[1], Olli Lahdenoja[1], Mojtaba Jafari Tadi[1,2*], Tero Hurnanen[1], Tuija Vasankari[3], Tuomas Kiviniemi[3], Juhani Airaksinen[3], Tero Koivisto[1], and Mikko Pänkäälä[1]**

[1]University of Turku, Department of Future Technologies, Turku, 20520, Finland
[2]University of Turku, Faculty of Medicine, Turku, 20520, Finland
[3]Heart Center, Turku University Hospital, Turku, 20520, Finland
[*]mojtaba.jafaritadi@utu.fi

## ABSTRACT

In this supplementary information material, additional details on classification performances are presented. Four types of data were considered for two-class, three-class, and four-class classification tasks. Kernel support vector machines (KSVM) and ranfom forests (RF) were employed for all classification tasks with and without majority voting. Performance metrics in this study include sensitivity, specificity, accuracy, and $F_1$ score. The results of classifications are also given in confusion matrices for each classifier separately.

## Supplementary tables for multiclass classifiers

### Two-class classification

Confusion matrices of the each classification type is given for random forest (RF) and Kernel support vector machines (KSVM) with and without majority voting as follows:

**Table 1.** RF confusion matrix for 2-class classification with and without majority voting

| RF-Without Majority Voting | | | | | | | | |
|---|---|---|---|---|---|---|---|---|
| | Normal | AFib | | Normal | PrePCI | | STEMI | PrePCI |
| Normal | 291 | 42 | Normal | 293 | 40 | STEMI | 239 | 62 |
| AFib | 32 | 552 | PrePCI | 73 | 197 | PrePCI | 100 | 170 |

| RF-With Majority Voting | | | | | | | | |
|---|---|---|---|---|---|---|---|---|
| | Normal | AFib | | Normal | PrePCI | | STEMI | PrePCI |
| Normal | 22 | 1 | Normal | 21 | 2 | STEMI | 17 | 4 |
| AFib | 1 | 39 | PrePCI | 5 | 16 | PrePCI | 8 | 13 |

**Table 2.** KSV confusion matrix for 2-class classification with and without majority voting

| KSVM-Without Majority Voting | | | | | | | | |
|---|---|---|---|---|---|---|---|---|
| | Normal | AFib | | Normal | PrePCI | | STEMI | PrePCI |
| Normal | 314 | 19 | Normal | 275 | 58 | STEMI | 240 | 61 |
| AFib | 29 | 555 | PrePCI | 48 | 222 | PrePCI | 107 | 163 |

| KSVM-With Majority Voting | | | | | | | | |
|---|---|---|---|---|---|---|---|---|
| | Normal | AFib | | Normal | PrePCI | | STEMI | PrePCI |
| Normal | 23 | 0 | Normal | 21 | 2 | STEMI | 16 | 5 |
| AFib | 1 | 39 | PrePCI | 4 | 17 | PrePCI | 8 | 13 |

Performance metrics for each two-class type are defined as accuracy, sensitivity, and specificity according to the

following Equations:

$$Sensitivity(SE) = \frac{\sum TP}{\sum TP + \sum FN} \tag{1}$$

$$Specificity(SP) = \frac{\sum TN}{\sum TN + \sum FN} \tag{2}$$

$$Accuracy(ACC) = \frac{\sum TP + \sum TN}{\sum TP + \sum FP + \sum FN + \sum TN} \tag{3}$$

where TP is true positives, meaning that disease case correctly identified as diseased, FN is false negatives meaning that disease case incorrectly identified as healthy, TN is true negatives meaning that healthy case correctly identified as healty, and FP is false positives meaning that healthy subject incorrectly selected as diseased case.

### Multi-class Classification Scoring

Classification scoring for multiclass case in this study was an $F_1$ measure, which is obtained as an average of

$$Normal : F_{1n} = \frac{2 * Nn}{\sum N + \sum n} \tag{4}$$

$$AFib : F_{1a} = \frac{2 * Aa}{\sum A + \sum a} \tag{5}$$

$$STEMI : F_{1m} = \frac{2 * Mm}{\sum M + \sum m} \tag{6}$$

$$PrePCI : F_{1p} = \frac{2 * Pp}{\sum P + \sum p} \tag{7}$$

The final scores are generated for each of the classification types as follows:

$$F_1 = \frac{F_{1n} + F_{1m} + F_{1p}}{3} \tag{8}$$

$$F_1 = \frac{F_{1n} + F_{1a} + F_{1m} + F_{1p}}{4}. \tag{9}$$

**Table 3.** Definition of parameters for scoring used in Eq. 8 and 9

| | Normal | AFib | STEMI | PrePCI | Total |
|---|---|---|---|---|---|
| Normal | Nn | Naf | Nm | Np | $\sum N$ |
| AFib | An | Aa | Am | Ap | $\sum A$ |
| STEMI | Mn | Ma | Mm | Mp | $\sum M$ |
| PrePCI | Pn | Pa | Pm | Pp | $\sum P$ |
| Total | $\sum n$ | $\sum a$ | $\sum m$ | $\sum p$ | |

**Table 4.** RF confusion matrix for 3-class classification with and without majority voting

| RF- Without Majority Voting | | | | | RF-With Majority Voting | | | | |
|---|---|---|---|---|---|---|---|---|---|
| | Normal | STEMI | PrePCI | Total | | Normal | STEMI | PrePCI | Total |
| Normal | 286 | 27 | 20 | 333 | Normal | 21 | 1 | 1 | 23 |
| STEMI | 6 | 246 | 49 | 301 | STEMI | 0 | 17 | 4 | 21 |
| PrePCI | 53 | 81 | 136 | 270 | PrePCI | 4 | 7 | 10 | 21 |
| Total | 345 | 354 | 205 | | Total | 25 | 25 | 15 | |

**Table 5.** KSVM confusion matrix 3-class classification with and without majority voting

| KSVM- Without majority voting | | | | | KSVM-With Majority Voting | | | | |
|---|---|---|---|---|---|---|---|---|---|
| | Normal | STEMI | PrePCI | Total | | Normal | STEMI | PrePCI | Total |
| Normal | 273 | 28 | 32 | 333 | Normal | 21 | 1 | 1 | 23 |
| STEMI | 2 | 237 | 62 | 301 | STEMI | 0 | 17 | 4 | 21 |
| PrePCI | 30 | 90 | 150 | 270 | PrePCI | 2 | 6 | 13 | 21 |
| Total | 305 | 355 | 244 | | Total | 23 | 24 | 18 | |

**Table 6.** RF Confusion matrix 4-class classification with and without majority voting

| RF- Without majority voting | | | | | | RF-With Majority Voting | | | | | |
|---|---|---|---|---|---|---|---|---|---|---|---|
| | Normal | AFib | STEMI | PrePCI | Total | | Normal | AFib | STEMI | PrePCI | Total |
| Normal | 276 | 20 | 12 | 25 | 333 | Normal | 21 | 1 | 1 | 0 | 23 |
| AFib | 46 | 471 | 48 | 18 | 583 | AFib | 4 | 33 | 2 | 1 | 40 |
| STEMI | 8 | 113 | 149 | 31 | 301 | STEMI | 0 | 7 | 12 | 2 | 21 |
| PrePCI | 30 | 71 | 22 | 147 | 270 | PrePCI | 3 | 4 | 1 | 13 | 21 |
| Total | 360 | 675 | 231 | 221 | | Total | 28 | 45 | 16 | 16 | |

**Table 7.** KSVM Confusion matrix 4-class classification with and without majority voting

| KSVM- Without majority voting | | | | | | KSVM-With Majority Voting | | | | | |
|---|---|---|---|---|---|---|---|---|---|---|---|
| | Normal | AFib | STEMI | PrePCI | Total | | Normal | AFib | STEMI | PrePCI | Total |
| Normal | 267 | 21 | 21 | 24 | 333 | Normal | 20 | 1 | 0 | 2 | 23 |
| AFib | 8 | 461 | 97 | 18 | 584 | AFib | 0 | 35 | 5 | 0 | 40 |
| STEMI | 1 | 74 | 185 | 41 | 301 | STEMI | 0 | 5 | 13 | 3 | 21 |
| PrePCI | 21 | 54 | 49 | 146 | 270 | PrePCI | 2 | 5 | 3 | 11 | 21 |
| Total | 297 | 610 | 352 | 229 | | Total | 22 | 46 | 21 | 16 | |
